# Advances in Solid-State Fermentation Technology for Oilseed Meal: Strain Selection, Fermentation Strategies, and High-Value Applications

**DOI:** 10.3390/foods15183177

**Published:** 2026-09-08

**Authors:** Jingyu Wei, Chenchen Yao, Musfira Akram, Xiaoai Wang, Sheeza Rasheed, Yuqing Duan, Dongyan Chen, Kai Hu, Wenlin Li, Haihui Zhang

**Affiliations:** 1 School of Food Science and Engineering, Jiangsu University, Zhenjiang 212013, China; 19503888764@163.com (J.W.); 19552156117@163.com (C.Y.); musfira14akram@gmail.com (M.A.); wangxaaa0129@163.com (X.W.); sheezarasheed078@gmail.com (S.R.); dongyan.chen@ujs.edu.cn (D.C.); kai.hu@ujs.edu.cn (K.H.); 2 Institute of Food Physical Processing, Jiangsu University, Zhenjiang 212013, China; 3 Oil Crops Research Institute, Chinese Academy of Agricultural Sciences, Wuhan 430062, China; wenlinli2005@163.com

**Keywords:** solid-state fermentation, oilseed meal, fermentation strategies, high-value applications, bioactive peptides, anti-nutritional factors

## Abstract

Oilseed meal, the primary by-product of oil extraction, is rich in protein, dietary fiber, and minerals, offering significant development potential. However, its application in high-value feed and food is severely restricted due to anti-nutritional factors, leading to resource waste and environmental issues. Solid-state fermentation (SSF) provides a green and efficient approach for the high-value utilization of oilseed meal. This review comprehensively discusses the entire process of strain selection, fermentation strategies, and application of active products in the SSF of oilseed meal. Regarding strain selection, *Bacillus* spp. degrade macromolecular proteins and inhibit microbial contamination through protease and antimicrobial peptide production. *Lactobacillus* spp. enhance flavor and safety by producing acids and flavor compounds. *Aspergillus* spp. decompose cell walls and degrade phytate using their cellulase and phytase systems. For fermentation strategies, mixed fermentation achieves functional complementarity, enzyme–fungus synergy enhances substrate conversion, segmented fermentation optimizes the microbial environment, and physical field assistance boosts enzyme activity, collectively improving fermentation efficiency and nutritional quality. In product applications, fermented oilseed meal serves as both high-quality protein feed and a source of functional peptides and active polysaccharides with antioxidant and immunomodulatory activities, showing potential for functional foods and biomedicine. In conclusion, SSF technology effectively degrades anti-nutritional factors, improving the nutritional value and adding value to oilseed meal, thus representing a key strategy for resource conversion. Future efforts should prioritize high-performance strain selection, intelligent process monitoring, and green preparation of active products to promote industrial application and sustainable development.

## 1. Introduction

Oilseed meal, the primary agricultural by-product generated during the oil extraction of oilseed crops, boasts a global annual output of tens of millions of tons, encompassing soybean meal (SBM), rapeseed meal (RSM), cottonseed meal (CSM), and palm kernel meal (PKM), among others [1]. Endowed with a rich array of nutrients including proteins, carbohydrates, dietary fiber, and minerals, oilseed meal serves as a superior plant protein source and is extensively employed in animal feed production. According to the forecast of the OECD-FAO Agricultural Outlook, global consumption of livestock products is expected to increase by 14% by 2030 compared to the period from 2018 to 2020 [2]. The increasing demand for animal products highlights the urgent need for feed resources [3]. Nevertheless, the high content of trypsin inhibitors in soybean meal impairs protein digestibility, while lectins induce digestive disorders [4], and free gossypol in cottonseed meal exerts adverse effects on animal growth and reproduction [5]; phytic acid and tannic acid in rapeseed meal form complexes with proteins, thereby compromising protein solubility and digestibility [6,7]. Furthermore, the anti-nutritional factors and harmful substances contained in the oilseed meal not only limit its wide application in the feed and food industries, but also result in a large amount of high-protein oilseed meal being used only as low-value fertilizers or simply composted, or even becoming waste, thus losing its potential for high-value processing and causing the waste of the nutritional value of the oilseed meal [8].

To enhance the efficient utilization of oilseed meal, a variety of technologies have been developed for the degradation of anti-nutritional factors (ANFs), encompassing physical, chemical, and biotechnological approaches [9]. Common physical processing techniques include heat treatment, extrusion, and micronization [10]. However, conventional methods like heating struggle to inactivate certain heat-stable ANFs; conversely, they may trigger protein denaturation, the formation of Maillard reaction products, and a reduction in nutrient digestibility [11]. Additionally, acid–base wastewater generated from chemical treatments poses environmental pollution risks.

Solid-state fermentation (SSF) technology has received extensive attention in recent years and is regarded as an appropriate method for converting agricultural industrial wastes and by-products into value-added products such as bioactive substances [12,13]. An effective method for extracting natural flavors from agricultural food wastes through SSF has also received considerable attention [14]. SSF technology can enhance the nutritional and functional properties of oilseed meal [15]; SSF of oilseed meal utilizes the enzymes produced during the growth and metabolism of microorganisms to degrade anti-nutritional factors and also generates active substances, thereby improving the nutritional quality of oilseed meal and enhancing its value-added applications. In the context of the circular economy, SSF may represent the most promising approach for obtaining valuable products from waste and by-products [16].

Although SSF technology has been widely applied in the processing of oilseed meal such as rapeseed meal and soybean meal, the existing research mostly focuses on a single aspect such as strain selection or process optimization, lacking a systematic integration. This review comprehensively summarizes the breakthroughs in key aspects such as strain selection and fermentation process optimization in SSF of oilseed meal. It reveals the transformation of SSF technology from traditional experience to precise fermentation, fills the academic gap in the theoretical integration of meal and residue SSF technology, provides a scientific basis for its transition from empirical parameter optimization to theoretical design guidance, and promotes the development of meal and residue SSF technology towards higher efficiency, higher precision, and more sustainable development.

This article adopts a narrative review format. The literature sources are from Web of Science, Scopus, and PubMed. The core keywords include solid-state fermentation, oilseed meal, strain screening, anti-nutritional factors, and high-value utilization. It mainly covers publications from the past decade. The selection is based on the titles, abstracts, and full texts, with priority given to peer-reviewed original articles and high-quality reviews. This review is limited to solid-state fermentation.

## 2. SSF Technology for Oilseed Meal

### 2.1. SSF Definition and Principles

**Figure 1:**
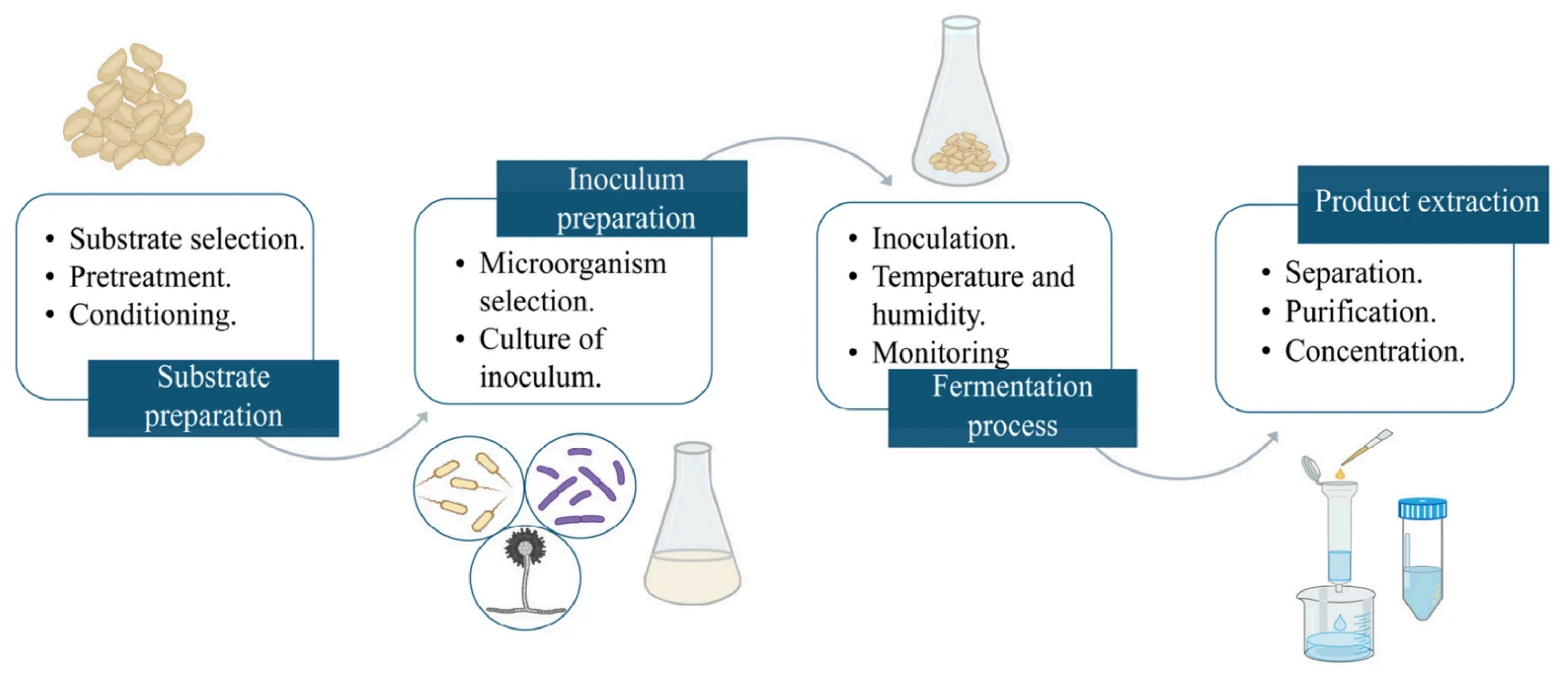
The process of oilseed meal SSF.

SSF is a biotransformation technology that uses solid substrates without free-flowing liquids as physical support and nutrient sources for microbial cultivation; it offers advantages such as low cost, low energy consumption, and high metabolic yield [17]. Compared with traditional submerged fermentation, SSF does not require a large amount of water medium and is more suitable for the high-fiber matrixes of oilseed meal [18]. Microorganisms can grow on the solid substrate to simulate their natural fermentation environment [19], and by secreting phytase, protease and cellulase, they can efficiently degrade the anti-nutritional factors in the oilseed meal, while generating small-molecule substances with biological activity [10]. The SSF process is shown in Figure 1. Therefore, microbial SSF is an ideal choice for improving the nutritional quality of oilseed meal and is used to produce many high-value enzyme preparations, bioactive compounds, and secondary metabolites [4,20].

### 2.2. The Respective Matrix Characteristics of Oilseed Meal and the SSF Method

#### 2.2.1. Soybean Meal

SBM is a by-product of extracting oil from soybeans. It offers several advantages, including a high protein content of approximately 40–50% and a well-balanced amino acid profile. Additionally, SBM serves as a widely utilized oilseed meal resource with significant value in various applications [21]. However, soybean meal has problems such as high protein denaturation [22], poor protein solubility [23], and unpleasant taste [24], and contains a large amount of anti-nutritional factors. Anti-nutritional factors such as phytic acid, lectins, tannins, saponins and protease inhibitors can interact with nutrients, thereby reducing their bioavailability [25], which limits the wide application of SBM in animal feed. Therefore, the development of efficient SBM value-added technologies not only enables the resource utilization of agricultural by-products, but also can prevent the adverse environmental impacts caused by the waste treatment process.

Traditional methods for improving the application quality of soybean meal mainly include heat treatment and enzymatic hydrolysis. Due to the poor heat resistance of anti-nutritional factors such as trypsin inhibitors, lectins and saponins, heat treatment can effectively reduce the content of anti-nutritional factors in SBM [26]. However, excessive heat treatment will affect digestibility and destroy amino acids through the Maillard reaction. In addition, proteases are capable of reacting with highly denatured proteins to break disulfide bonds and improve the extractability of these proteins [27]. Proteases are relatively expensive and may produce bitter peptides, which can adversely affect the flavor of the oilseed meal [28]. Therefore, improving the quality and efficiency of soybean meal raw materials through microbial SSF is a promising method. Research shows that the anti-nutritional factors in SBM, such as trypsin inhibitors, phytic acid and lectins, are reduced by 70% to 90% after microbial SSF [29,30,31]. In the traditional SSF process, sterilization is usually carried out with steam or high pressure before fermentation to eliminate bacteria in the SBM substrate and equipment, and then one or more purebred microorganisms are inoculated for fermentation. Nevertheless, these sterilization procedures can cause the degradation of the original protein [32]. To solve this problem, it is possible to inoculate microorganisms for fermentation without the need for pre-sterilization of the SBM by screening local strains for natural fermentation of soybean meal [19].

#### 2.2.2. Rapeseed Meal

Rapeseed meal (RSM), as a by-product of rapeseed oil extraction, is the second largest source of plant protein in the world after soybean meal. It is mainly composed of protein, fiber and minerals, rich in sulfur amino acids, and has a good balance of essential amino acids [33,34]. However, RSM contains a relatively high amount of anti-nutritional factors such as glucosinolates, lignocellulose, phenolic compounds and phytic acid, which can interfere with the absorption and digestion of proteins and bring a bitter or astringent taste to food [35], thus limiting the high-value-added conversion of rapeseed meal [36,37].

In order to enhance the effective utilization rate of rapeseed meal, it is necessary to carry out detoxification and anti-nutritional factor removal treatment. The traditional processing method of rapeseed meal reduces the nutritional value of protein and the content of effective lysine, seriously affecting the functional activity of rapeseed polypeptides [36]. At present, two primary high-value processing strategies are employed for RSM. The first is microbial solid-state fermentation (SSF), which mainly enriches the nutritional value of agricultural by-products by degrading cellulose and lignin fibers and eliminating anti-nutritional factors. The second involves enzymatic pre-treatment of RSM using industrial-grade enzymes. For instance, pre-treatment of RSM with fiber-degrading enzymes significantly increased the concentrations of glucose and fructose, thereby enhancing the utilization rate of nutrients in rapeseed meal [38]. Moreover, the bacterial strains used in solid-state microbial fermentation may lack some extracellular hydrolases, resulting in incomplete removal of anti-nutritional factors. Therefore, the use of enzyme and microbial synergistic fermentation to improve the nutritional value of RSM has become a promising method. For instance, through the combined fermentation of enzymes and microorganisms, the content of small-molecule proteins in RSM increased by 81.7%, while the content of glucosinolates decreased by 30.06% [39].

#### 2.2.3. Cottonseed Meal

Due to factors such as the global economy, extreme weather, the area of cultivated land and its nature as a shared resource for both humans and animals, the supply of soybean meal is becoming increasingly limited. Therefore, some other raw material components, such as cottonseed meal (CSM), are widely used in feed to replace soybean meal in the diet. This can not only ensure food security, but also promote the sustainable development of animal husbandry and aquaculture [40]. CSM is the solid residue left after extracting oil from cottonseeds. It is rich in crude protein (34–40%), crude fiber (11%), vitamin B, organophosphorus and other nutrients [41]. It can be used as a high-quality protein feed resource to alleviate the shortage of protein feed resources.

However, due to the fact that free gossypol (FG) may significantly affect animal development, leading to tissue damage and a decline in reproductive performance, the application of cottonseed meal in animal husbandry and aquaculture is restricted [42]. Excessive intake of gossypol can cause several clinical symptoms in animals, including anorexia, metabolic disorders, and even death [43]. Therefore, considering the health and safety of both humans and animals, it is necessary to eliminate the toxic effects of FG in animal feed.

Traditional treatment methods include physical methods such as heat treatment, liquid cyclone separation and air classification, as well as chemical methods such as solvent extraction to remove gossypol. However, these methods present problems such as nutrient damage, high cost and pollution [27,44]. The safe processing or environmentally friendly transformation of cottonseed meal is crucial for the sustainable development of cotton [45]. Microbial SSF is a promising strategy for detoxifying cotton by-product gossypol [46]. By using the microorganism SSF method, not only can FG in CSM be removed, but macromolecular proteins can also be degraded, thereby enhancing the nutritional value and palatability of cottonseed meal [47].

#### 2.2.4. Others

With the development of agriculture, traditional oilseed meal has been unable to meet the feed demands of the livestock industry. As a result, various alternatives to traditional meal, such as palm kernels [48], flaxseeds [49], and camellia oleifera seeds [50], have emerged. They contain fatty acids and active substances. However, their application is limited due to the presence of anti-nutritional factors [48,51,52]. In addition, there are many agricultural by-products such as peanut meal and walnut meal that have similar development bottlenecks. To break through the application limitations of non-traditional meal, enzymatic hydrolysis combined with SSF by microorganisms is an ideal approach. Existing studies have shown that the effect of enzyme–bacteria co-fermentation of feed components is superior to that of bacteria or enzymes alone. The former demonstrates obvious advantages in enhancing the nutritional value of feed and feeding performance [53].

## 3. Selection of SSF Strains for Oilseed Meal Products

### 3.1. Applicable Strains

Different microorganisms play indispensable roles in the fermentation process with their unique enzyme systems and metabolic products. Therefore, screening and understanding the functional characteristics of key microorganisms suitable for oilseed meal fermentation is the basis for designing and optimizing the fermentation process.

**Figure 2:**
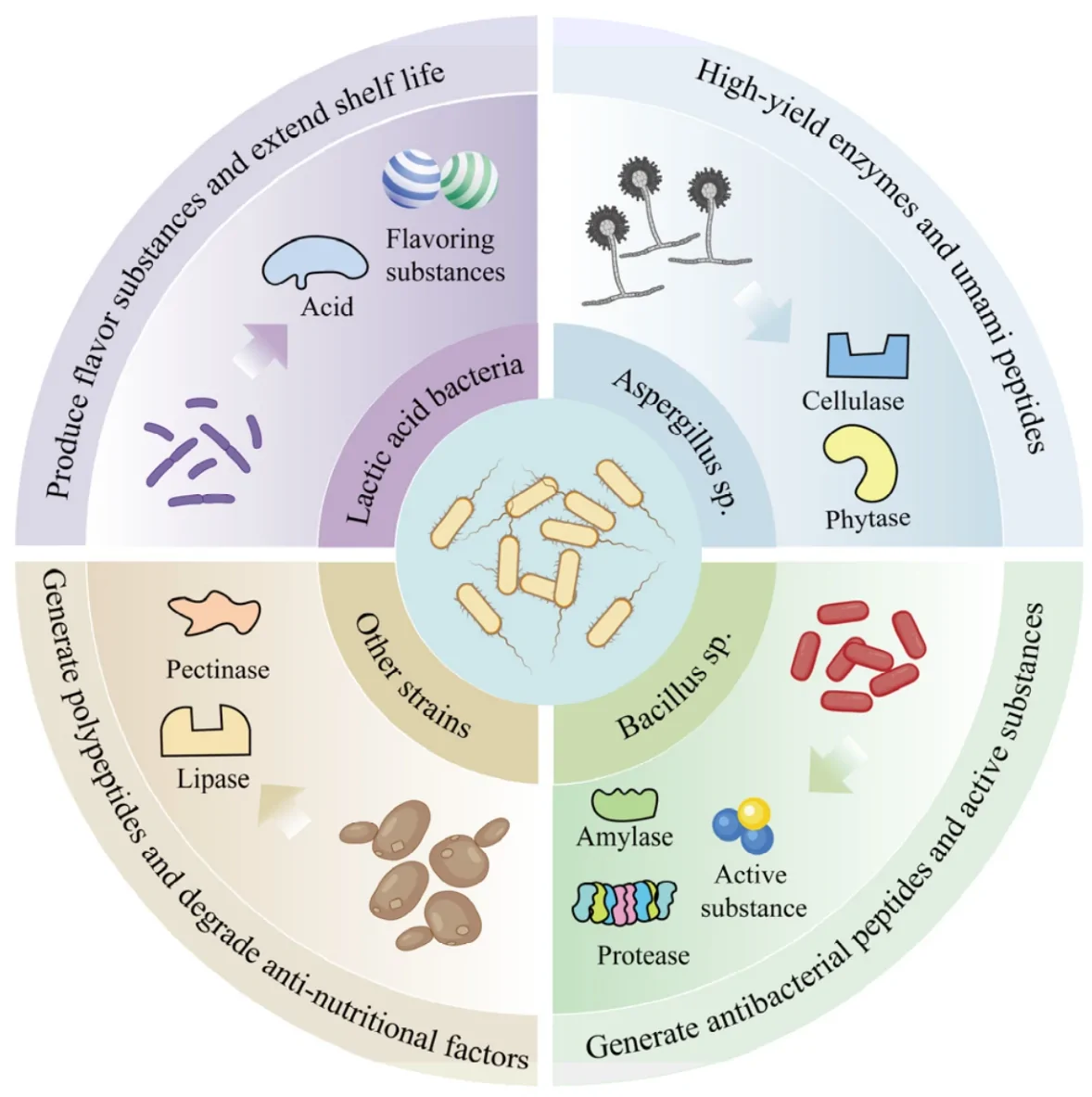
Functional characteristics of fermentation strains.

The three most widely used and functionally representative types of microorganisms in oilseed meal fermentation include *Bacillus subtilis*, lactic acid bacteria, and molds (mainly *Aspergillus niger* and *Aspergillus oryzae*) [54,55,56]. The main functional characteristics of *Bacillus subtilis* include secreting strong proteases and amylases, forming spores for easy storage, and oxygen consumption, creating a favorable environment for anaerobic bacteria such as lactic acid bacteria. The main functional attributes of molds include the robust secretion of proteases, phytases, cellulases, and other hydrolytic enzymes, enabling efficient degradation of soy protein and subsequent generation of umami-enhancing amino acids. The main functional characteristics of lactic acid bacteria include rapidly producing acid to inhibit pathogenic bacteria such as Salmonella and Escherichia coli, being able to produce antibacterial substances, and producing flavor substances. The characteristics of the fermentation strains are shown in Figure 2. This section will introduce the main functions of *Bacillus subtilis*, lactic acid bacteria and *Aspergillus* fungi in the SSF of oilseed meal.

#### 3.1.1. *Bacillus* sp.

Among the genus *Bacillus*, *Bacillus subtilis* has been studied in depth for decades. The complete genomic sequence has been fully decoded, and the biological functions of most genes and their encoded proteins have been well characterized. *Bacillus subtilis* has been classified as a generally recognized safe bacterium [57]. *Bacillus subtilis* can secrete proteases to catalyze the hydrolysis of peptide bonds, breaking down proteins into amino acids and peptides [58]. It can also secrete cellulase to break down repetitive glucose units in the straight chains linked by β-1,4-glycosidic bonds in carbohydrates. It can also secrete amylase and lipase to break down large-molecule substances such as polysaccharides and fats into small-molecule components like monosaccharides and fatty acids [59]. This enzymatic capacity significantly enhances nutrient utilization and reduces anti-nutritional factors. Therefore, it is widely used in animal feed production [60].

In addition to secreting various enzymes to promote the decomposition of macromolecular substances, *Bacillus subtilis* can also secrete antibacterial substances such as subtilosin A and iturins, which can inhibit the growth of miscellaneous bacteria during the fermentation process [61]. In addition, it can secrete active substances such as vitamin K, vitamin B2, and polysaccharides. Vitamin K plays a significant role in the treatment of osteoporosis, cardiovascular diseases, neurological disorders, and cancer, among other conditions. Vitamin B2 is involved in the oxidation-reduction and metabolism of cells and is related to the metabolism of nucleic acids, proteins, and fats, maintaining the normal functions of cells [62]. Active polysaccharides can inhibit the proliferation of HepG2 cells [63].

Based on the powerful enzyme secretion capacity of *Bacillus*, it can efficiently decompose macromolecular substances. It also inhibits miscellaneous bacteria by secreting antimicrobial peptides to ensure the smooth progress of fermentation. At the same time, it can also synthesize value-added components such as vitamins and active polysaccharides by itself, enhancing the comprehensive functionality and economic value of fermented products.

#### 3.1.2. Lactic Acid Bacteria

Lactic acid bacteria play a key role in the fermentation of oilseed meal. Plant proteins usually have a strong and unpleasant raw material flavor, including bitterness and astringency, which may limit their application [64]. *Lactobacillus* can form flavors through the accumulation of metabolites during carbohydrate metabolism, protein breakdown and amino acid metabolism, fat breakdown and fatty acid metabolism [65,66]. Flavor components such as flavorings, aldehydes and ketones produced by pyruvate metabolism can provide the aroma of cooked cherries and roses for fermented products [67]. The accumulation of glutamic acid and proline in protein and amino acid metabolism can enhance umami and sweetness [68]. Derivatives such as alcohols, ketones and esters in the process of fat metabolism may also bring rich flavors to fermented foods.

In addition to enhancing flavor, *Lactobacillus* fermentation can improve the nutritional value, functionality, rheology and texture of grain products. Its metabolites, extracellular polysaccharides, can affect its viscosity, water-holding capacity and stable exopolysaccharide–protein complex network, thereby improving the texture characteristics of the final product [66]. *Lactobacillus* can also acidify, extend shelf life or degrade other anti-nutrients [69], and inhibit the growth and metabolism of harmful microorganisms [70,71]. *Lactic acid bacteria* employed in SSF can enhance the sensory and functional properties of raw materials [72], and are primarily utilized in the production of protein hydrolysates [73]. At present, *Lactobacillus* fermentation is widely used in the food, medical and feed industries [74].

In conclusion, *Lactobacillus* can transform raw materials with unpleasant flavors into high-quality feed with a sour and fragrant smell and an attractive taste through complex metabolism. By rapidly generating acid, an environment unfavorable for the survival of harmful bacteria is created, providing natural preservation and ensuring the stability and safety of the product. This improves the physical texture of the product by generating polysaccharides and other means, and enhance its nutritional value.

#### 3.1.3. *Aspergillus* sp.

The most commonly used Aspergillus species are *Aspergillus niger* and Aspergillus oryzae. The production rate of *Aspergillus niger* mycotoxins is relatively low. By carefully selecting the strains and controlling the fermentation conditions, the risk of mycotoxin contamination can be effectively reduced, ensuring its safe application in bioprocessing. It is the most frequently used fungus in SSF research [55,56,75]. *Aspergillus niger* primarily produces cellulase, hemicellulase, protease, and phytase. These enzymes are capable of breaking down complex carbohydrate and protein molecules, thereby reducing anti-nutritional factors present in meals.

*Aspergillus oryzae* strains have attracted much attention in the food industry due to their rapid colony growth, abundant spores and high enzyme activity [76]. *Aspergillus oryzae* mainly produces protease and amylase, and is often used to ferment soybeans to make soy sauce and other products, endowing soy sauce with rich and complex flavors. During the fermentation process, regulating the growth and metabolism of Aspergillus oryzae to secrete more proteases is an important strategy in enhancing the umami intensity of soy sauce. Therefore, some researchers used Aspergillus oryzae to ferment SBM to isolate umami peptides [77], exploring the development and application of umami-enhancing peptides from the hydrolyzed products of SBM fermentation.

#### 3.1.4. Other Strains

In addition to the commonly used strains mentioned above, yeasts can also be used for oilseed meal fermentation, such as *Candida tropicalis* and *Meyerozyma guilliermondii*. Research has found that the adaptation of *Candida tropicalis* to gossypol mainly works by activating the antioxidant defense system to alleviate the oxidative stress response induced by gossypol [78]. Deng et al. fermented rapeseed meal with *Schizochytrium*; under optimal fermentation conditions, the contents of polypeptides and total free amino acids increased by 47.0% and 71.63%, respectively. The maximum degradation rates of glucosinolates, oxazolethione, and isothiocyanates reached 61.36%, 43.68%, and 55.47%, respectively [79]. A comparison of the nutritional quality improvement of fermented oilseed meal by different strains is shown in Table 1.

In order to further enhance fermentation efficiency, in recent years, various fermentation strains have been continuously explored. The process mainly involves isolating original strains from environments rich in microbial communities, and then, through cultivation and performance testing, selecting strains with specific target functions, such as high production of certain enzymes or efficient degradation of toxins.

Some researchers isolated the strain M-2, which produces β-glucosidase, from cow dung and applied it to the fermentation of flaxseed meal. The decolorization rate of cyanoglycoside reached 89%, and the crude protein increase reached 44%. These results provide a theoretical basis for the removal of cyanogenic glycosides in flaxseeds and the comprehensive utilization of flaxseed cakes, and offer new ideas for degrading anti-nutritional factors in oilseed meal through fermentation [49]. A strain with strong enzyme-producing ability, *Bacillus coagulans* S17, was also isolated from feces and screened. This strain was applied to the fermentation of cottonseed meal and the fermentation conditions were optimized. After the fermentation, the content of FG in the SBM decreased from 923.80 mg/kg to 167.90 mg/kg, and the detoxification rate was 81.83%. At the same time, the crude protein content of the bacterial body increased from 47.98% to 52.82%, and the spore production of strain S17 reached 1.68 × 1010 CFU/g dry matter. A feasible and effective process for degrading gossypol in cottonseed meal was established [88,89].

### 3.2. Breeding of High-Performance Strains

**Figure 3:**
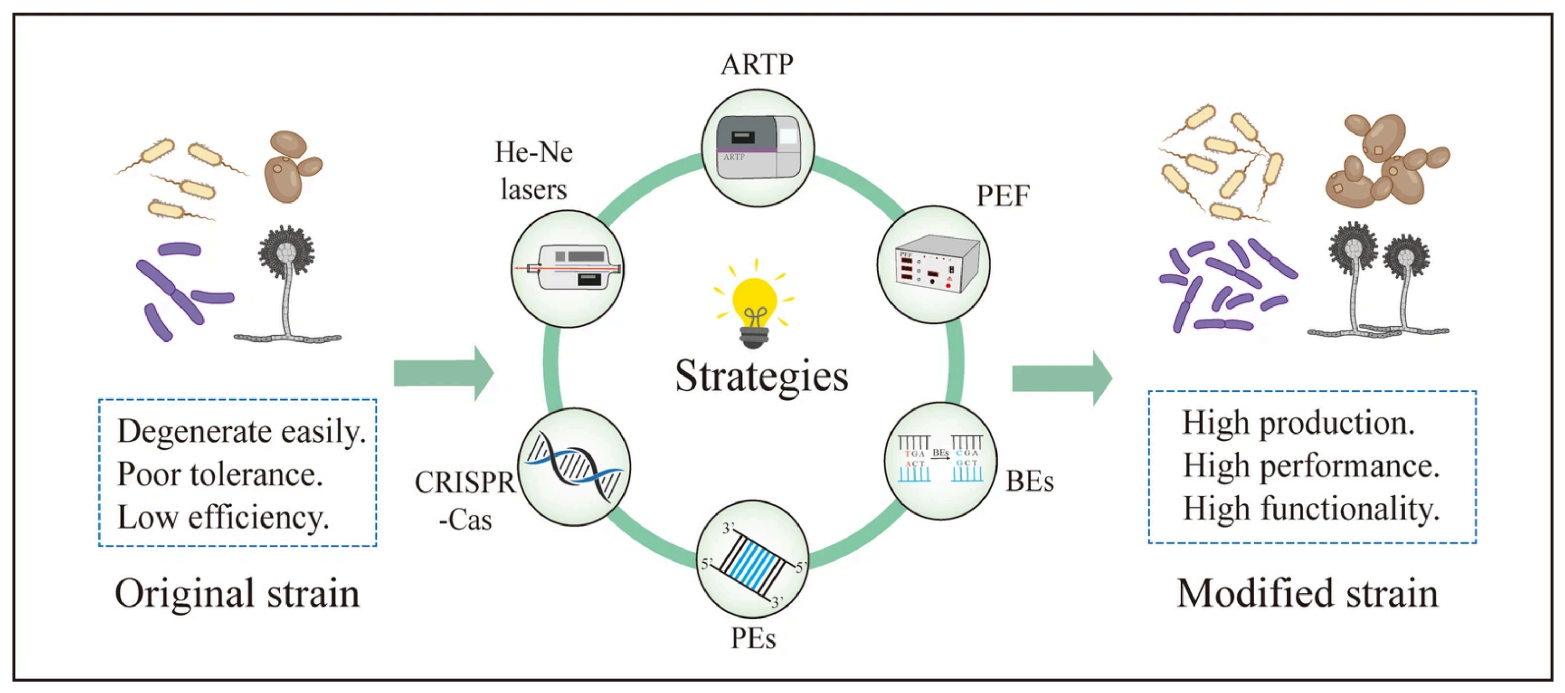
Isolation of high-performance strains for oilseed meal SSF through mutagenesis techniques (ARTP: atmospheric and room-temperature plasma; PEF: pulsed electric field; BEs: base editors; PEs: prime editors).

To obtain superior fermentation microorganisms capable of effectively degrading anti-nutritional factors, releasing functional peptides, and enhancing antioxidant capacity during solid-state fermentation (SSF), strains are subjected to mutagenesis and then the mutant strains with desirable phenotypic traits are screened for fermentation to boost efficiency [37]. The strain mutagenesis strategy in SSF is shown in Figure 3.

#### 3.2.1. Random Mutagenesis

The treatment of microbial cells mainly involves random mutagenesis through physical and chemical methods, introducing any mutations into the microbial genome. The most widely used mutagens in traditional mutagenesis projects are alkylating agents [90], and the most commonly used physical mutagenesis methods mainly include ultraviolet rays, gamma rays, X-rays, etc. In recent years, methods such as pulsed electric fields, lasers, and plasma have also been used to randomly mutate bacterial strains [90]. Currently, there are extensive studies on the use of physical methods for breeding of fermentation bacterial strains. Random mutagenesis is used to enhance the characteristics of the strains, and then the metabolic patterns of microorganisms are further explored through genome sequencing.

Ultraviolet irradiation mutagenesis studies have been conducted on isolated *Bacillus licheniformis* strains to further enhance their fermentation capacity and increase the activity of the fermentation products (peptides) [91]. Recent studies have successfully applied combinatorial mutagenesis and whole-genome sequencing to modify *Bacillus* species, significantly enhancing their protease production capacity and stability during the fermentation process [92] and subsequently significantly enhancing the activity of protein hydrolytic enzymes. ARTP (atmospheric and room-temperature plasma) technology was used to mutagenize the starch-fermenting bacteria BS5582 and the MI-fermenting strain 3.042, and the acid protease activity of the strains increased by 45.03% and 54.7%, respectively [20,24]. The obtained mutant strain *Bacillus licheniformis* BS5582 was used for soy sauce fermentation, and the amino acid nitrogen and free amino acid contents in the soy sauce increased by 18.06% and 32.54%, respectively [20]. This provides guidance for obtaining high-enzyme-activity strains through ARTP technology mutagenesis.

#### 3.2.2. Gene Editing

Homologous recombination, plasmid and expression vector-based strategies, and CRISPR-Cas genome editing represent three pivotal technologies in the field of biotechnology [18]. Ongwl et al. improved the enzyme production via gene modification of the selected *Bacillus subtilis* F6 by various regulatory elements, including promoters, ribosome binding sites and signal peptides [83], thereby increasing its ability to produce mannanase and making the hydrolysis of palm powder and other fiber substrates more efficient. In recent years, various Cas homologs with useful additional properties and their variants have been identified and utilized for gene editing. Moreover, new tools for precise gene modification, such as base editors (BEs) and prime editors (PEs), have greatly expanded the application scope of gene editing and have been applied in multiple research fields [93,94]. CRISPR-Cas-related technologies have also been extended to enable targeted DNA modification by introducing functional genetic elements, thereby facilitating precise genome editing, programmable regulation of gene expression, and site-specific epigenetic remodeling [95]. The CRISPR-Cas9 gene editing tool can precisely modify the genome [96]. Using the guide RNA sequence, Cas9 recognizes the NGG PAM sequence on the DNA and cuts to form a blunt-ended double-strand break, thereby creating an opening that allows for insertion, deletion or substitution of the gene sequence. CRISPR-Cas12 can recognize PAM sequences rich in T and cut to produce a sticky end. CRISPR-Cas13 can directly recognize RNA targets, cut RNA and have an accompanying RNAase activity. CRISPR-Cas gene editing technology can improve the strain by modifying genes that control metabolism, stress resistance and metabolite production [97].

Random mutagenesis methods such as ARTP, laser, and mutagens are simple-to-operate and cost-effective high-throughput approaches. However, they have low controllability and require a lot of work for the screening of mutant strains and the detailed elucidation of mutagenesis mechanisms. CRISPR-Cas gene editing technology can generate double-strand DNA breaks at specific genomic locations, introducing precise changes through the cell repair mechanism. However, it has relatively low throughput and requires the design of guide RNA, as well as specific requirements for delivery and the cell repair mechanism.

## 4. Improving Oilseed Meal Nutritional Quality Through Diverse SSF Strategies

**Figure 4:**
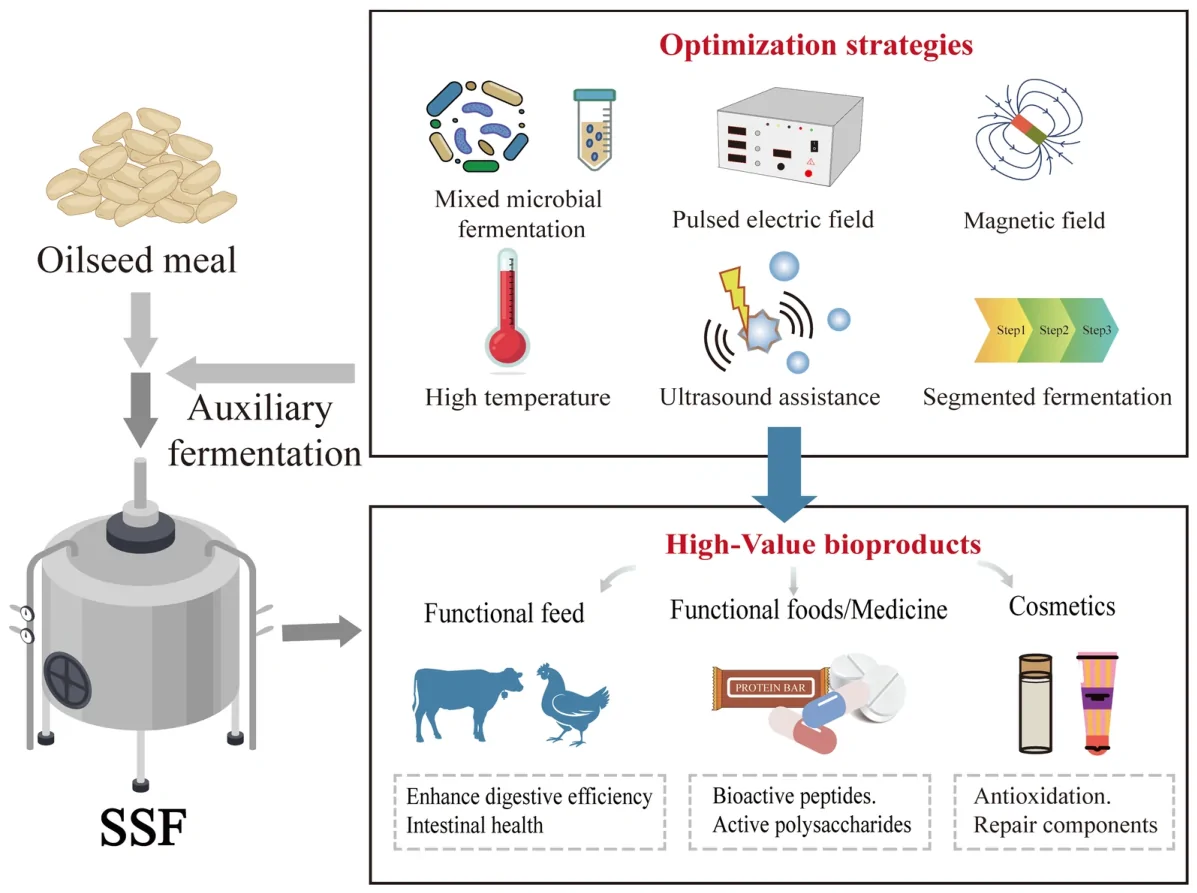
SSF strategies for oilseed meal and application of its active products.

SSF is an efficient biological conversion technology; however, its performance is significantly influenced by various fermentation parameters. The SSF method is also constantly innovated and optimized, forming various fermentation strategies with specific advantages and applicable scenarios. These methods achieve efficient conversion and value enhancement of oilseed meal resources through different microbial combinations, process conditions, and technical auxiliary means (Figure 4).

### 4.1. Mixed Bacteria SSF

Through single-strain fermentation, the anti-nutritional substances in the oilseed meal can be effectively reduced, and the accumulation of nutrients and active molecules in the oilseed meal can be increased. Compared with single cultures, multiple microorganisms can produce a wider range of metabolites, generate various enzymes, and promote the biotransformation of anti-nutritional factors and macromolecular materials [98].

To explore better fermentation effects, based on the different fermentation actions and effects of various strains, the co-fermentation of multiple strains of microorganisms can improve the nutritional quality of the meal and the meal by-product. During the SSF process, adding mixed fungi such as Aspergillus oryzae, Aspergillus Iberia, and Aspergillus Niger can produce more cellulase and xylanase and enhance bioactive substances, thereby increasing the nutritional value of the meal and improving the by-products of animal feed [86]. The same process of co-fermentation with *Rhizopus acerophyllus* and *Mucor racemosus* was applied to wheat germ cakes. After the fermentation, the activities of peptides, soluble phenolic acids, amino acids and other substances in the wheat germ cakes were all enhanced, providing a new technique for the fermentation of wheat germ cakes by fungi [99]. The synergistic fermentation of rapeseed meal by *Saccharomyces cerevisiae* and *Brevibacterium* sp. can effectively reduce the content of anti-nutritional factors in rapeseed meal and increase the protein content. After 24 h of fermentation by *Saccharomyces cerevisiae* and *Brevibacterium* sp., the contents of glucosinolates and 3-butenyl isothiocyanate in the fermented rapeseed meal decreased by 51.60–66.04% and 55.21–63.39%, respectively [100].

In addition to exploring the synergistic fermentation of the same type of bacteria, synergistic effects between different bacterial strains are also being continuously discovered. For example, when using *Bacillus subtilis*, *Lactococcus lactis*, and *Candida tropicalis* to ferment RSM, through high-throughput sequencing analysis of microbial diversity in the fermented rapeseed meal, it was found that mixed bacterial fermentation can inhibit the growth of miscellaneous bacteria, with these strains better able to carry out metabolic activities [101]. Wang et al. mixed and fermented SBM with strains of *Bacillus cereus*, *Enterococcus faecalis* and *Staphylococcus aureus*, and the small peptides increased from 4.48% to 47.4%, while β-protein decreased from 232.11 μg/mL to 32.89 μg/mL, a reduction of 85.83% [102]. Using *Lactobacillus delbrueck* and *Bacillus subtilis* to ferment rapeseed meal, the content of thioglycosides in rapeseed meal decreased from 64.558 μmol/g to 3.473 μmol/g, achieving a high degradation rate [28]. Using *Bacillus subtilis* GYB6, *Saccharomyces cerevisiae* NJ1, and *Bacillus* Y8 for SSF of rapeseed meal, the contents of crude protein, amino acids, and peptides in the fermented rapeseed meal significantly increased, while glucosinolates, phytic acid, crude fiber, and tannins significantly decreased [103].

In summary, SSF methods with mixed bacteria are mainly divided into co-fermentation with the same type of microorganism and co-fermentation with different types of microorganisms. Synergistic fermentation with the same type of microorganism takes advantage of the complementarity of enzyme systems secreted by different bacteria to improve the quality of oilseed meal through synergistic fermentation. Synergistic fermentation with different types of microorganisms is more common and has a more comprehensive effect. SSF with mixed bacteria can significantly enhance nutritional value and increase the content of crude protein, peptides and amino acids. Highly efficient degradation of anti-nutritional factors significantly improves the content of functional substances and antioxidant activity.

### 4.2. Synergistic SSF by Enzyme–Bacteria Mixture

Fermentation and enzymatic hydrolysis can effectively alter the phenolic content in grains, fruits, and plant oil processing by-products [104,105]. The synergistic fermentation of cellulase and strains can effectively improve the digestibility and extraction rate of protein in meal. When cellulase is mixed with *Bacillus subtilis*, *Saccharomyces cerevisiae*, and *Nigella sativa mold* for fermentation of rapeseed meal, under the optimal fermentation conditions, the co-fermentation of multiple strains and cellulase significantly reduces the anti-nutritional factors in the steam–pressure fermentation process, and the solubility, digestibility, and extraction rate of protein increases by 466.80%, 45.56%, and 816.44%, respectively [106]. Adding exogenous proteases to the fermentation system can increase the content of soluble protein [102]. When exogenous proteases and strains were co-fermented with rapeseed meal, it was found that the soluble protein content reached 78 g/100 g [107]. Song et al. used high-efficiency enzyme preparations, which mainly consisted of lipase, *hemicellulase*, *yeast*, *lactic acid bacteria*, and starch-decomposing bacteria and digestive bacteria, to ferment rapeseed meal. The enzyme and microorganisms jointly promoted the conversion of macromolecular substances during the fermentation of rapeseed meal [38].

They work together to efficiently and purposefully detoxify anti-nutritional factors, optimize nutritional components, transform functional substances, and improve the overall fermentation quality. This technology is one of the key green biotechnologies for efficiently utilizing renewable resources such as by-products of grain processing and meal to produce high-value-added feed or food ingredients.

### 4.3. SSF of Oilseed Meal in Stages

Due to the metabolic activities of microorganisms during fermentation, which influence key environmental parameters such as pH, dissolved oxygen levels, moisture content, and internal substrate temperature, fermentation progress may eventually reach a stage where conditions become suboptimal for further microbial growth. Consequently, targeted adjustments to critical growth parameters, including pH and moisture content, are necessary to sustain efficient and continuous fermentation [108].

It was found that staged fermentation can significantly improve the nutritional quality of the oilseed meal. Staged fermentation, such as conducting aerobic fermentation first and then anaerobic fermentation, can further decompose large-molecule nutrients. After aerobic fermentation with *Bacillus subtilis* for 12 h, followed by anaerobic fermentation with a combination of *yeast*, *Lactobacillus plantarum*, and *Lactococcus lactis*, under the optimal fermentation conditions, the contents of small peptides, amino acids, and organic acids all significantly increased [109]. Dai et al. studied the effects of single-stage and two-stage SSF of Lichen *Thermophilic Bacillus* YYC4 on soybean meal [108]. The two-stage SSF significantly improved the interface properties and enhanced the nutritional value and antioxidant activity of soybean meal. By conducting staged fermentation on cottonseed meal with *Bacillus natto* N-2 and the fungus WST-M1, after the first stage of SSF with *Bacillus natto* N-2 at 30 °C for 120 h and the second stage of SSF with the *fungus* WST-M1 at 38 °C, the contents of water-soluble protein and trichloroacetic acid-soluble protein in the cottonseed meal significantly increased, while the free gossypol decreased to 653.26 ± 16.25 mg/kg [110].

Segmented fermentation can significantly enhance nutritional and functional value, more efficiently degrade anti-nutritional factors, and improve the physicochemical properties of the product. The core advantage of this method lies in creating an appropriate fermentation environment for each type of functional microorganism, thereby maximizing their capabilities and ultimately achieving an improvement in the nutritional value, functional activity, and detoxification effect of the fermented oilseed meal.

### 4.4. Physical Processing Assists SSF

The high efficiency of enzymes can significantly enhance the fermentation performance of oilseed meals. However, enzymatic reactions typically require mild operating conditions, making enzymes prone to inactivation and difficult to obtain; direct procurement is also costly. Consequently, researchers have been actively exploring novel fermentation techniques that are low in energy consumption, easily accessible, and technically feasible to assist in the fermentation of oilseed meals. These approaches not only improve the antioxidant properties of the fermented products but also effectively enhance their functional characteristics [111].

Pulsed electric field (PEF)-assisted fermentation is an emerging hybrid technology that integrates microbial metabolism with electroporation-induced cell activation. This process involves applying direct current pulses between two electrodes to generate reversible pore formation in the membrane, which promotes microbial growth and facilitates metabolite release [112]. In food fermentation systems, PEF-assisted fermentation of watermelon juice has been shown to increase lactic acid yield and promote the release of bioactive compounds in fermented beverages [113]. The fermentation seed liquid treated with PEF was used to ferment rapeseed meal, which increased the protease activity, polypeptide content and phenolic content, thereby enhancing the functional and structural properties of rapeseed meal [37].

Magnetic field-assisted fermentation is a recently developed approach that regulates microbial growth, metabolic synthesis, and food quality through multi-level interactions with cells, such as enzyme activity, ion transport, and gene expression, thereby influencing fermentation outcomes [114,115]. Exposure to static or time-varying magnetic fields has been shown to activate specific metabolic pathways and increase the production of beneficial metabolites. Adding a low-intensity magnetic field during the SSF of rapeseed meal can effectively alter the structure and enhance the nutritional and protein digestibility of the fermented rapeseed meal [116,117].

Conventional fermentation processes are typically conducted at ambient temperatures, which coincides with the optimal growth range for many contaminating and pathogenic microorganisms, posing a risk of fermentation failure. In contrast, high-temperature SSF selectively employs thermophilic microorganisms under elevated temperature conditions (commonly 50–65 °C or higher), overcoming some inherent limitations of mesophilic fermentation, but utilizing a high-temperature environment can effectively inhibit the growth of harmful bacteria. For instance, researchers isolated a thermophilic protease-producing strain, RM-2, identified as a thermophilic *Geobacillus stearothermophilus*, and applied it to the high-temperature SSF of unsterilized rapeseed meal for peptide production. The peptide yield following RM-2 fermentation reached 9.67% [9]. Increments of soluble and crude protein by 277.19% and 18.63% and decrement of harmful TI by 61.05% in SBM were achieved using thermophilic SSF by *Bacillus licheniformis* YYC4 [118].

Apart from adding low-intensity magnetic fields and high-temperature SSF, the use of ultrasonic assistance in SSF of oilseed meal has also been widely welcomed. Ultrasonic assistance utilizes acoustic cavitation, which can disrupt cell structures and activate biochemical pathways [119,120]. Cavitation can trigger chemical effects, such as the ultrasonic decomposition of water, generating hydroxyl radicals and hydrogen peroxide, which may regulate the activity of microbial enzymes [121,122]. It has been reported in the literature that, by using ultrasound to prepare the fermentation seed solution and then conducting SBM fermentation with this seed solution, ultrasound treatment can significantly increase its biomass, cell membrane permeability and metabolic capacity [123,124], as well as enhance the content of active substances in the fermentation products [125,126]. It was found that ultrasonic treatment can strengthen the fermentation process and increase the content of soluble proteins and soluble dietary fibers in SBM [127]. It is reported that ultrasonic treatment can also significantly promote the release of amino acids and enhance the flavor of the fermentation substrate [128,129,130].

By utilizing physical fields to stimulate microorganisms or modify the material structure, it is possible to go beyond the limitations of traditional fermentation and achieve better fermentation results. This has achieved significant effects such as enhancing fermentation efficiency, increasing the yield of target products, improving the physical and chemical structure of the oilseed meal, and enhancing the functional activity of the product. It provides a new technical path for the production of high-quality fermented oilseed meal products. The advantages and limitation of different fermentation strategies are shown in Table 2.

### 4.5. Monitoring of the Fermentation Process

During the fermentation process, the fermentation environment changes as the microorganisms grow and metabolize, altering factors such as pH, oxygen consumption, and temperature [133]. If the fermentation conditions change and are not adjusted in time, which is not conducive to the growth of microorganisms, some harmful substances may be produced. Timely monitoring of the fermentation process and adjustment of parameters can better enhance the fermentation efficiency and improve the nutritional quality of the oilseed meal. In recent years, online monitoring systems for changes in the fermentation process and physical and chemical indicators have been established [134,135,136], such as establishing a prediction model for color changes and pH changes during fermentation, providing ideas for online monitoring of the fermentation process [137,138].

It is reported that spectral technology can be used to collect information during the fermentation process to establish a model for monitoring the fermentation process [139,140]. Xu et al. collected Raman spectra of microorganisms in SSF of vinegar and established a database, further established a Raman-fermentation stage regression model, and combined it with machine learning to provide a new method for the monitoring of SSF of vinegar [141]. Based on attenuated total reflection mid-infrared spectroscopy and colorimetry, combined with chemometrics, efficient and comprehensive monitoring of the fermentation process of yeast-producing Candida albicans can be achieved [142]. Online monitoring technology based on near-infrared spectroscopy has also been widely studied [143,144,145]. Based on near-infrared spectroscopy and partial least squares discriminant analysis, bread fermentation state detection technology can specifically detect un-fermented dough, fermented dough, and over-fermented dough [146,147]. In the SSF of oilseed meal, near-infrared technology can also be utilized, combined with the stoichiometric method, to monitor the key process parameters during the fermentation process, enabling real-time measurement and adjustment of the SSF [148,149].

In addition, the exploration of biosensors for monitoring the fermentation process has also been widely studied [150]. Using data fusion strategies to integrate electronic nose, electronic tongue, and colorimeter sensors to monitor the fermentation process of agaricus bisporus [151], using a nanostructured platinum integrated microsensor array to enhance the characterization of pH, temperature, conductivity, and glucose system in the fermentation process [152], and integrating sensing into intelligent learning to establish a multivariate statistical process control model, which can provide effective quality control strategies for food fermentation processes and ensure the consistency, stability, and controllability of product quality [153].

## 5. SSF Products of Oilseed Meal and Their Application Potential

After the oilseed meal is fermented, it is commonly used as feed for livestock. When adding fermented cottonseed meal to the diet of laying hens, it was found that feeding with fermented cottonseed meal can improve eggshell quality, promote intestinal health and reduce serum and egg yolk cholesterol levels [154].

In addition, during the oilseed meal fermentation process, significant secondary metabolites are produced [155], and these bioactive compounds have various biological activities, including preventing chronic diseases such as cardiovascular diseases, diabetes and cancer [156]. The extraction of active substances from fermented oilseed meal has also become a research hotspot in the transformation of biological resources [157], especially on the active peptides produced after fermentation.

### 5.1. Active Protein

Active proteins are biologically active organic compounds formed by the dehydration condensation of amino acids. Their amino acid sequences are encoded by genes and can be classified into four grades based on structure. They are widely present in animals, plants and microorganisms. The active protein has immunomodulatory functions, antioxidant effects and antithrombotic effects. Its medicinal functions cover antithrombosis, skin repair and immune regulation, and it is applied in cell therapy, skin care product research and development and the food industry.

The bioactive proteins generated through microbial fermentation of oilseed meal are predominantly extracellular enzymes secreted by the fermenting microorganisms, including proteases, cellulases, and phytases. These enzymes hydrolyze the raw materials of oilseed meal to produce active polypeptides or proteins and degrade anti-nutritional factors. Researchers extracted functional proteins from cottonseed meal for study and found that hydrolyzed DES-extracted protein (CSMP-EH) exhibits enhanced DPP-IV-inhibitory activity (IC_50_ = 1.949 mg/mL) [158]. Reddy et al. investigated the ability of *Bacillus licheniformis*, *Acinetobacter pittobacter* and *Aspergillus niger* to produce enzymes through fermentation of Koji seed meal under different culture conditions [159]. Purified proteases possess decontamination power, gel layer hydrolyzability and coagulability, and can serve as a new enzyme source for various dairy and biotechnology industries. By using *Aspergillus tubingensis* NKBP-55 to ferment coconut meal, this enzyme preparation can degrade complex agricultural residues, such as sugarcane bagasse and straw [160].

During the fermentation process, microorganisms secrete a large amount of extracellular enzymes, which are themselves highly active proteins and the core driving force for the transformation of oilseed meal.

### 5.2. Bioactive Peptides

Bioactive polypeptides are discrete small protein fragments that, in addition to their role as food, also possess specific biological functions. In microbial fermentation, the metabolism of microorganisms themselves can also generate some bioactive peptides. This method often increases the yield and enhances the activity [54,161].

Peptides exhibit diverse biological activities, such as antioxidant properties, blood pressure-lowering activity, antibacterial activity, immunomodulatory activity, hormonal and signaling molecule functions, and promotion of mineral absorption [162,163]. The active peptides obtained through enzymatic digestion of plant proteins have extensive nutritional and health-promoting properties [164]. Microbial fermentation can also produce bioactive peptides that can prevent certain diseases [165]. Some studies have used specific strains for fermentation to produce antihypertensive peptides. After fermentation of oilseed meal, the extraction rate of peptides can be increased, and antioxidant activity and anti-aging effects can be effectively enhanced [166]. Fermentation of soybean meal with *Bacillus* KN36D isolated five new antioxidant peptides as microbial resources for developing other fermented foods, providing a reference for recovering bioactive compounds from by-products in similar biochemical environments [167]. Co-fermentation of cotton meal with *Lactobacillus* LLK-XR1 and acid protein hydrolysis enzyme can extract active peptides [168,169,170], and through molecular docking, molecular dynamics simulation and cell experiments, the antioxidant properties of five peptides were verified, which can effectively alleviate oxidative stress damage caused by hydrogen peroxide in macrophages. In addition, active peptides can play a crucial role in reducing oxidative stress by inhibiting specific enzymes responsible for increasing oxidative stress [171]. Studies have used ultrasound-assisted preparation of active peptides from flaxseed meal to obtain three antioxidant peptides; these peptides displayed scavenging activity against DPPH radicals (IC_50_ = 0.520, 1.190, and 1.036 mM) and hydroxyl radicals (IC_50_ = 4.909, 6.471, and 7.076 mM) [172]. Another study indicated that the active peptides obtained from fermented rapeseed meal with *Bacillus licheniformis* DY145 have the potential to alleviate intestinal inflammation and enhance the high-value utilization of rapeseed meal [91].

### 5.3. Active Polysaccharides

Polysaccharides, also known as glycans, are high-molecular-weight carbohydrates formed by the connection of glycosidic bonds through the dehydration condensation of multiple monosaccharide molecules. The active functions of polysaccharides include immunomodulatory function, anti-tumor activity, antioxidant and anti-aging, hypoglycemic and lipid-lowering, and intestinal health regulation [173].

Studies have shown that when using *Bacillus subtilis* to ferment soybean meal, the structure of the polysaccharides is significantly different. The results of extracting the polysaccharides and conducting in vitro fermentation further indicate that the fermented soybean meal polysaccharides significantly promote the number of intestinal symbiotic bifidobacteria and lactobacilli in pigs, and enhance the microbial functions for metabolizing fructose and mannose. These findings elucidate the microbial genomic mechanisms during the fermentation process, revealed the potential regulatory role of FSPs on the intestinal microbial flora, and expand the utilization of SBM [80]. Other researchers have engineered Escherichia coli to use palm nut meal as a carbon source to decompose mannans to produce hyaluronic acid. Hyaluronic acid has been further applied in the fields of food, cosmetics, and clinical medicine [174].

## 6. Economic and Environmental Benefit Analysis

Compared with submerged fermentation, the advantages of SSF lie in its similarity to the natural habitat of microorganisms, higher productivity, lower water consumption, less use of chemicals, less generation of waste gas, and lower energy consumption [175]. SSF uses cheap agricultural waste as the substrate, significantly reducing raw material costs, and through microbial metabolism during the fermentation process, it can produce high-value enzyme products. Research has shown that using SSF to produce cellulase for second-generation ethanol production has economic benefits. When the enzyme activity is above 12 FPU/g.d.s and there is sufficient substrate supply, the cost of cellulase produced by SSF is between 1.88 and 3.51 US dollars per 10^6^ FPU (2014-year value, Brazilian sugar-energy industry context). The off-site commercial cellulase cost under the same market and year context is estimated at 7.8–12.5 US dollars per 10^6^ FPU for second-generation ethanol biorefinery applications [176]. Wei Han et al. conducted a technical and economic analysis of a new biogas production process combining SSF and dark fermentation from kitchen waste. The unit biogas production cost is 2.29 US dollars per cubic meter, lower than the market price of 2.7 US dollars per cubic meter (values based on Chinese market economic parameters) [177]. The biogas production process using SSF is economically feasible. Gao et al., through SSF of pre-treated straw, showed that the utilization rates of cellulose and hemicellulose in the pre-treated straw reached 95.25% and 46.88% respectively, and the conversion rates of ethanol and xylose were 93.00% and 29.19% respectively [178]. This result provides a theoretical basis for the industrial application of high-solid enzyme and alkaline co-pre-treatment to achieve more energy-efficient and sustainable development.

The SSF technology promotes circular economy and efficient resource utilization by reducing raw material costs and energy costs and improving product added value [179], thereby enhancing economic benefits. In addition, by fermenting agricultural by-products and food industry by-products, converting them into useful products that would otherwise need to be treated or incinerated, waste disposal costs can be reduced. Therefore, effectively handling waste can reduce environmental pollution, and is in line with the sustainable development trend.

## 7. Challenges and Future Works

### 7.1. Challenges

Although SSF technology has significant advantages in the application of oilseed meal fermentation, there are several fundamental challenges that limit its wider industrial implementation. After long-term passage and cultivation, the strains may exhibit genetic instability, leading to a decline in key functions such as enzyme activity, thereby affecting product quality. Currently, most methods for modifying the strains utilize random mutagenesis, aiming to select high-performance strains, which are uncertain, and the screening workload is large. Moreover, the fermentation process is influenced by multiple factors, and currently, industrial production mainly relies on offline sampling and testing, with feedback lag and difficulty in achieving real-time and precise control. Near-infrared spectroscopy, electronic nose and electronic tongue, among others, have made progress in online monitoring technologies, but they are limited by substrate characteristics and the anti-contamination ability of sensors, and various solid-state fermentation methods more effectively promote the nutritional quality of oilseed meal and the yield of active substances; however, due to the limitations of substrate characteristics and the anti-contamination ability of sensors, the emerging auxiliary solid-state fermentation methods are mostly at the laboratory-scale stage and further large-scale experiments still need to be combined with industrial production. For the fermentation products, although their feed value has been confirmed through animal experiments, the process of separating and purifying the active substances is complex and costly, which limits their high-value applications in the fields of food and medicine. Special safety-related considerations may arise if fermented oilseed meal is targeted for human-food uses. Risks mainly include residual anti-nutritional factors, potential mycotoxin contamination and biogenic-amine formation during SSF; accordingly, only food-grade microbial strains should be employed. Regulatory requirements vary geographically, food-grade fermented oilseed meal ingredients generally need novel-food assessment or GRAS notification, while feed-oriented standards are not directly transferable to human-food matrices. Unified safety specifications for such food-ingredient products remain limited.

### 7.2. Future Works

Future research efforts should focus on the modification of strain gene editing, the in-depth application of online monitoring systems, the amplification experimental tests of emerging fermentation processes, and the expanded application of active substances in fermentation products. Excellent strains with high enzyme production and specific metabolite production should be selectively bred through gene editing tools such as CRISPR-Cas. At the same time, multi-omics technologies should be combined to analyze the metabolic mechanism of microbial colonies. The fermentation process requires timely monitoring and feedback. Non-destructive detection technologies such as near-infrared spectroscopy and hyperspectral imaging, as well as biomimetic sensors, should be combined to obtain real-time dynamic information on the chemical composition of materials, microbial quantity, and flavor substances, and machine learning algorithms should be introduced to integrate historical data and online signals to achieve multivariate predictive regulation. Furthermore, the emerging fermentation strategies should undergo scale-up experiments to investigate their economic feasibility in the industrial production process. For the fermentation products, in addition to high-quality feed, the application of the functional components of fermented oilseed meal in health products, medicines, cosmetics and other fields should be further expanded. At the same time, the development of green and efficient extraction technologies should be carried out to reduce the purification costs.

## 8. Conclusions

This article comprehensively reviews the research progress of SSF technology for oilseed meal, covering the entire process from strain resource selection and innovation of diversified fermentation strategies to the high-value application of the products. It summarizes the microbial resources represented by *Bacillus subtilis*, *lactic acid bacteria*, and *Aspergillus* through advanced strategies such as mixed fermentation, synergistic bacteria-enzymes, segmented fermentation, and physical field assistance, which can efficiently degrade anti-nutritional factors such as free gossypol, glucosinolates, and phytic acid, significantly increase the protein solubility and small peptide and free amino acid contents of oilseed meal, and enrich active components such as antioxidant substances. This not only converts oilseed meal from potential waste into a high-quality protein source for feed, but also opens up a path to obtain high-value products such as bioactive peptides, functional polysaccharides, and high-value enzyme preparations from it. Future research directions should focus on overcoming the current bottlenecks in the industrialization process: (1) Utilizing gene editing technology to create efficient engineered strains and deeply analyze the interaction mechanisms between microorganisms and complex substrates in the SSF system. (2) Promoting the intelligence of fermentation equipment and the application of online monitoring technologies in order to achieve precise control of the process. (3) Developing economically feasible separation and purification schemes for active products and establishing a scientific product efficacy evaluation system to expand their applications in feed, food, agriculture, and even medical and health care and other broader fields. SSF technology is not only a key strategy for enhancing the nutritional value of meal and meal by-products, but also an important means for implementing circular economy and achieving sustainable agricultural development. Promoting in-depth research and industrial application of this technology has important strategic value and practical significance for ensuring global food security, reducing the reliance of the livestock industry on traditional protein feed, and promoting the development of environmentally friendly agriculture.

## Figures and Tables

**Table 1:** Comparison of fermented oilseed meal effects among different strains.

Specific Strain	Substrate	Increased Nutrients	Reduced ANFs	Reference
*B. subtilis* BS12	SBM	Peptides: 12.11% Amino acids: 41.90%	Cellulose 38.83%	[80]
*B. subtilis* ED-3-7	SBM	Acid-soluble protein: 342.61%	Urease: 90.10%	[29]
*Bacillus* *licheniformis*	SBM	Peptide yield: 68.58% Protein conversion rate: 82.27%	Trypsin inhibitor activity: 59.17%	[81]
*B. subtilis* 67	RSM	Crude fat: 48.85%	Crude fiber: 10.68%	[82]
*B. subtilis* 21095	RSM	Protease activity: 196.31% Peptide: 49.41%	-----	[37]
*B.subtilis* F6	PKM	Crude protein: 1.3%	Neutral detergent fiber: 36.4%	[83]
*Lactobacillus mucosae* LLK-XR1	CSM	Peptides: 46.25%	Free gossypol: 85.63%	[84]
*Lactobacillus reuteri*	SBM	Acid-soluble protein: 4.85% Lactic acid: 225.83%	β-Conglycinin: 33.92% Glycinin: 58.08%	[85]
*Aspergillus oryzae*	SBM	Umami-enhancing peptides EA: 232.0%	-----	[24]
*Aspergillus niger*	PKM	Crude protein: 44.3%	Neutral detergent fiber: 9.6%	[86]
*Treptomyces* SCUT-3	CSM	Soluble protein: 35.5%	Free gossypol: 77.8%	[87]
*Meyerozyma guilliermondii* WST-M1	CSM	Acid-soluble protein: 78.51%	Free gossypol: 74.70%	[27]
*Candida tropicalis* ZD-3	CSM	-----	Free gossypol: 92%	[78]
*Schizochytrium* ATCC 20888	RSM	Polypeptide: 47.0% Total free amino acids: 71.63%	Glucosinolates: 61.36% Oxazolidinethion: 43.68% Isothiocyanates: 55.47%.	[79]

**Table 2:** The principle and fermentation effect of the SSF strategy for oilseed meal.

Fermentation Strategy	Core Principle	Main Advantages	Limitations	Typical Effects
Single strains	Enzymatic hydrolysis action	High controllability. The process is simple.	Single-function. Low stability.	The yield of the polypeptide increased by 47.74%. The activity of trypsin inhibitor decreased by 76.11% [131].
Mixed strains	Microbial synergy	Comprehensive functions. Effect enhanced. Ecological stability.	The structure of the microbial community and its metabolic products are complex.	The content of polypeptides significantly increased [101]; the antigen protein decreased by more than 85% [102].
Enzyme-coordinated	Exogenous enzyme preliminary hydrolysis, microbial deep metabolism	Highly efficient. Targeted and precise. Effect optimization.	The cost of enzymes is high and they are prone to inactivation.	The solubility of proteins has significantly increased [107].
Segmented	Create growth environments for different microorganisms	Optimization of fermentation environment. Process refinement.	The process is complex.	Improved the antioxidant activity and protein structural properties of the product [108].
Physical-assisted	Utilize physical fields to stimulate microbial activity or modify the structure of substrates	Activate microbial metabolism. Improve the quality of the product.	High cost and poor adaptability	Ultrasound assistance increased the peptide content from 79.7 g/kg to 128.8 g/kg [132]. Pulsed electric field enhanced protease activity and phenolic content [37]. High-temperature fermentation directly utilized unsterilized raw materials, with a peptide yield of 9.67% [9].

## Data Availability

No new data were created or analyzed in this study. Data sharing is not applicable to this article.
